# Sustainable binary/ternary blended mortars with recycled water treatment sludge using fly ash or blast slag: Characterization and environmental-economical impacts

**DOI:** 10.1007/s11356-024-32175-7

**Published:** 2024-01-30

**Authors:** Ceren Kina

**Affiliations:** Department of Civil Engineering, Faculty of Engineering and Natural Sciences, Malatya Turgut Ozal University, Malatya, Türkiye

**Keywords:** Water treatment sludge, Ground-granulated blast furnace slag, Fly ash, Rheology and strength, Environmental impact and cost

## Abstract

Water treatment sludge (WTS) is produced daily and disposed of as hazardous material. It would be advisable to use locally available waste products as supplementary cementitious materials that ensure to be disposed of without harming the environment. As a novelty, this research investigated the potential of using recycled WTS with fly ash (FA) and ground-granulated blast furnace slag (BFS) as ternary blended binders. Thus, it can provide an economical solution and alleviate the adverse environmental effects of excessive production of wastes and cement production. Within this scope, the mortars with 0–30 wt% replacement of cement with modified WTS (MWTS) were produced as binary blend, and also, they were combined with FA/BFS as ternary blended binders. Therefore, optimum utilization of waste products into the mortar in terms of rheological, mechanical, durability, microstructural properties, and environmental-economical aspects was examined. Adding 10% recycled WTS as binary caused higher strengths with lower porosity measured by the mercury intrusion porosimeter test and denser microstructure, as revealed by XRD patterns and SEM results. However, the drawbacks of using recycled WTS, in terms of rheological parameters and environmental-economical aspects, were suppressed by adding FA/BFS with comparable strength values. Specifically, cost, CO_2_ footprint, and embodied energy were reduced by combining 10% MWTS with FA by 8.87%, 37.88%, and 33.07%, respectively, while 90-day compressive and flexural strength were 5.1% and 5.32% lower. This study developed a feasible solution to use recycled MWTS by obtaining more eco-friendly and cost-effective cement-based materials.

**Graphical Abstract:**

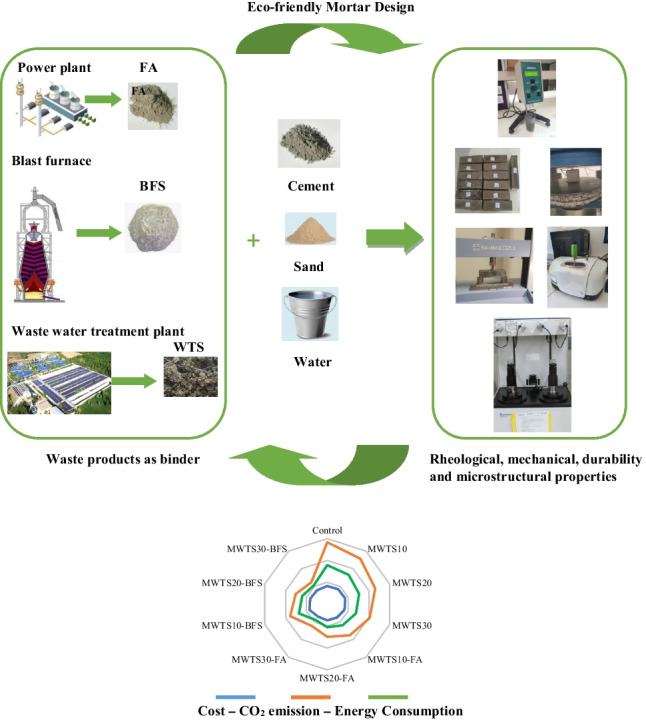

## Introduction

Increasing demand for products and services due to population growth causes the expansion of industrial activity, which in turn increases material consumption. The concrete industry is one of the areas where natural resources are used the most, and its growth causes significant environmental impacts related to the use of raw materials such as the release of large greenhouse gas emissions during the production of cement. The manufacturing process of cement releases more than 2 Gt carbon dioxide annually (Wei and Cen 2019), and 12–15% of the world’s total industrial energy is consumed (Nidheesh and Kumar 2019). Not only to reduce the environmental impacts caused by cement production, but also due to the shortage of natural resources, the development of environmentally friendly, cost-effective, and low-carbon building materials has become a topic of widespread concern (Turk et al. 2017; Qaidi et al. 2021; Hamada et al. 2022). In this context, the use of waste by-products including silica fume (SF), ground-granulated blast furnace slag (BFS), and fly ash (FA) as partial substitutions for Portland cement (PC) has started to attract attention worldwide (Diniz et al. 2022; Singh et al. 2023; Wang et al. 2023). SF is commonly used in concrete as a blended SF cement or in the form of water-based slurry and causes some benefits and drawbacks (Banar et al. 2022). FA is a by-product from the combustion of pulverized coal and is widely used separately or as a component of blended cement to improve some properties of concrete (Siddique 2010). BFS is obtained as waste in the production of pig iron in the blast furnace and provides important enhancements in mortar/concrete production (Özbay et al. 2016).

Wastewater treatment plants (WWTPs) are infrastructure facilities through which water purification is provided in most cities around the world. The water treatment process includes sedimentation, coagulation-flocculation, screening, chemical disinfection, and granular filtration (Bonton et al. 2012). After the stages of these processes, a by-product called water treatment sludge (WTS) is produced, which consists of a variety of inorganic and organic substances (Pestana et al. 2016). The residuals of the coagulation-flocculation process include consumed coagulants as well as suspended and colloidal impurities, which are removed from the liquid phase in the filtration process (Ahmad et al. 2021). The waste sludge consists of organic matter, solid particles, and aluminum or iron metal oxides. Population growth induces an increase in water consumption, which means more sludge is produced by WWTPs. Large amounts of WTS have become a global problem. Wei et al. (2020) showed that China generated 7.808 Mt of sludge per year. Besides, the disposal of WTS reached 43.500 t/year in Australia, while it was 131.000 t in the UK (Finlay 2015; De Carvalho Gomes et al. 2019). Therefore, the processing and utilization of WTS have become an urgent problem to be solved worldwide. In some underdeveloped countries, WTS is thrown into a drainage system or a river, which threatens human health (Ahmad et al. 2016), while it is utilized as agricultural fertilizers, industrial chemicals, and construction materials in some developed countries (Doudart de la Grée et al. 2018; Maierdan et al. 2020). Landfilling of WTS is another common method of disposal practiced in many cities. However, this method is no longer practical due to the difficulty in finding landfill sites and operating landfill costs. There is also some strict legislation that prohibits the disposal of this waste in a landfill especially in Europe (Ahmad et al. 2016).

The WTS includes a lot of pollutants, pathogens, and heavy metals so it can be categorized as a hazardous material (Godoy et al. 2019). The high-temperature treatment is a way to reduce the amount of WTS, and also, it causes to destroy pathogens, oxidize organic matter, and immobilize heavy metals but generate a high quantity of solid waste. Therefore, in order to recycle or reuse WTS, some sustainable alternatives should be investigated.

The application of WTS in industrial processes and building materials hs become preferable due to its availability as a raw material and its wide potential (De Carvalho Gomes et al. 2019). Currently, many studies have been performed using WTS in ceramic products, especially partial replacement of clay on fired bricks (Benlalla et al. 2015; Erdogmus et al. 2021; Amin et al. 2022; Harja et al. 2022). Furthermore, some attempts have been also made to utilize WTS as aggregates for cement-based composites (de Oliveira Andrade et al. 2018; Chen et al. 2022). However, the usage of WTS in these products is not sufficient for the reuse or recycling of a large amount of WTS. PC is the most widely used cementitious material, and its 1 t of production requires 2.5 t of raw material and generates 5–8% of the total carbon dioxide emission (Rashid et al. 2019; Yıldırım et al. 2021). Therefore, the reuse/recycling of WTS to be used as a partial replacement for PC will provide its large volume application.

Previous studies have shown that WTS has a clay-like mineral composition (Świerczek et al. 2018) as well as a chemical composition similar to cement clinker (Ebert et al. 2021), suggesting the potential for WTS to be used as a supplementary cementitious material. In the study by Gomes et al. (2020), 10% of PC was replaced by raw WTS, and it caused a 34.9% reduction in 28-day compressive strength with regard to the paste with no WTS. However, Godoy et al. (2019) identified an important finding for more efficient and effective use of WTS. They found that high-temperature calcination of raw WTS can provide an active pozzolanic effect and also immobilize heavy metals and reduce pathogens. The research of He et al. (2021) also indicated that the use of 10% calcined (modified) WTS as a replacement for PC enhanced the 90-day compressive strength, while 20% and 30% modified WTS caused the reduction. Besides, it was noted that the compactness of the microstructure of the mortar specimens was improved by the use of 10% modified WTS. Ahmad et al. (2018) reported that the replacement of PC by modified WTS increased the water demand to achieve consistency, and the usage of WTS up to 20% would provide a beneficial reuse. Frias et al. (2013) used heat-treated calcined kaolinite-based WTS and said that a 15% PC replacement ratio satisfied mechanical and physical specifications in current European cement codes. Gastaldini et al. (2015) found that the substitution of WTS for cement at percentages of 10%, 15%, 20%, 25%, and 30% improved the compressive strength ranging from 3–30%, depending on the water-binder ratio. In the study by Ruviaro et al. (2021), it was concluded that replacing up to 20% PC with calcined WTS caused almost equivalent compressive strength compared to the plain paste and did not significantly influence the fresh properties. Spat Ruviaro et al. (2023) also found from another study that the incorporation of 30% WTS and 15% filler as a replacement of cement caused only 10% lower compressive strength than the plain cement paste. Besides, Hagemann et al. (2019) found that the usage of 15% WTS increased the compressive strength, while for higher WTS content (15–28%), the compressive strength gain was less pronounced.

### Research significance

The increased emphasis on sustainable development and environmental protection in the construction industry has increased the use of supplementary cementitious materials. In this perspective, the achievement of desired workability, strength, and durability properties by incorporating different waste products is a widely common research issue in the literature. However, there are several gaps related to the environmental and technical feasibility of using WTS as a replacement for PC. Besides, although cost-effective cement-based materials have been already developed using industrial by-products including FA and BFS through optimizing the mix design in the literature, the cost of cement-based materials is still high. In this perspective, the use of recycled WTS is promising to develop more cost-effective and eco-efficiency composite. Based on the literature survey, some cement mortar mixtures were developed through recycling WTS (as binder) (Frias et al. 2013; Ahmad et al. 2018; Godoy et al. 2019; He et al. 2021; Ruviaro et al. 2021). On the other hand, the literature survey indicated that there is no research that examined the incorporation of calcined WTS with other waste products as a binder into cementitious composites. It points out the necessity of research on the effects of the use of modified WTS (MWTS) with some wastes including FA and BFS as ternary blended binders on the performance and composition of composite materials as well as to examine the economical and environmental impacts. Thus, as a novelty, the aim of this study is to examine the feasibility of using these waste products as ternary blends into mortar. The potential of using MWTS as a binary blend into mortar and using MWTS with the addition of FA or BFS as a ternary blend into mortar was investigated in terms of rheology, microstructural characterization, mechanical performance, porosity, and environmental and economical impacts. Despite several studies investigating the use of WTS in cement-based composites as mentioned above, more attention has been given to the mechanical and microstructural properties. In the literature, there have been only two researches (Ruviaro et al. 2021; Spat Ruviaro et al. 2023) which evaluated the rheological parameter. As a second novelty, in the current study, the rheological properties of binary and ternary blended mortars with MWTS were investigated to establish the feasibility of using these wastes in terms of fresh performance. Besides, compressive and flexural strengths were attained for mechanical properties, and porosity was measured via the mercury ıntrusion porosimeter (MIP) test. Meanwhile, fourier transform ınfrared spectroscopy (FTIR) analysis, X-ray diffractometer (XRD), and scanning electron microscopy (SEM) were used to obtain the effects of the incorporation of waste products on hydration products and microstructure of mortars. Moreover, environmental and economical evaluations were evaluated by considering the cost, carbon dioxide footprint, and embodied energy. Through this research, new sights were generated to reveal the interaction of MWTS with PC, FA and BFS, which is crucial to promote recycling/reuse of sludge and produce eco-friendly and cost-effective sustainable cement-based material.

## Materials and testing methods

### Raw materials

In the production of the mortars, the following binders were utilized:

- Ordinary CEM I 42.5R Portland cement (PC) conforming to TS EN 197–1
- Fly ash (FA) classified in class F based on ASTM C618
- Ground-granulated blast furnace slag (BFS) supplied from a local steel manufacturing plant
- Water treatment sludge (WTS) obtained from the Waste Water Treatment Plant at Malatya, Türkiye

**Fig. 1:**
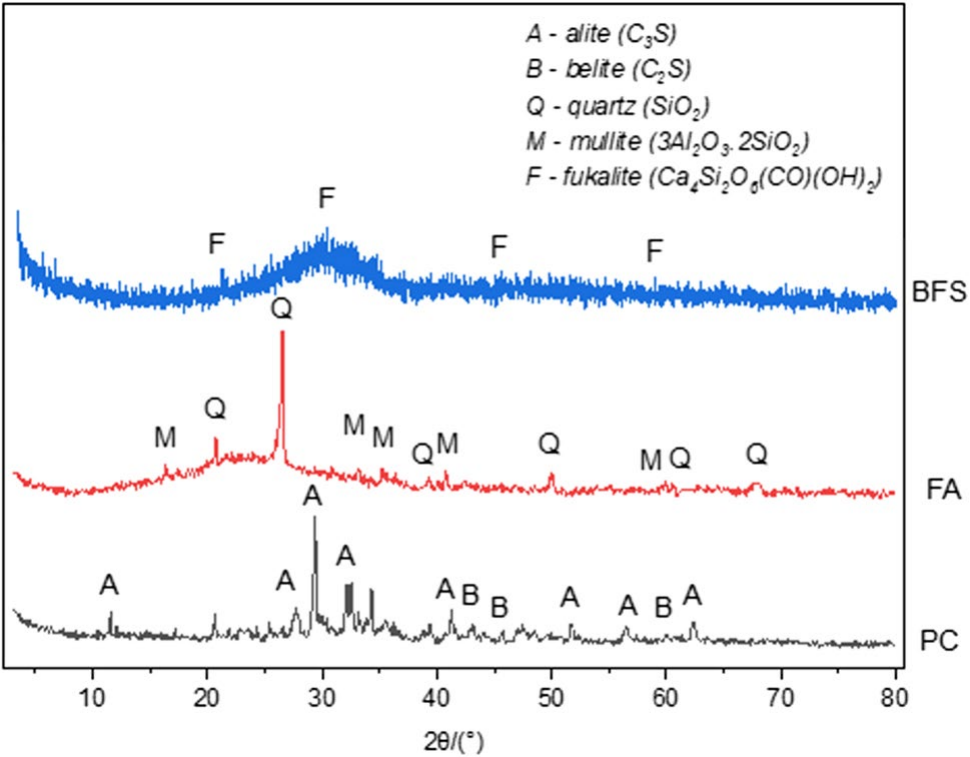
XRD patterns of the binders

**Fig. 2:**
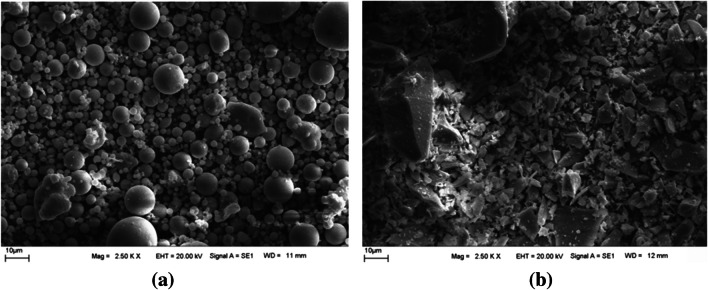
SEM images of (**a**) FA and (**b**) BFS

The characteristics of PC, FA, and BFS are summarized in Table 1, and their XRD and SEM images are given in Figs. 1 and 2, respectively.

**Table 1:** The chemical and physical characteristics of binders

	Binders		
	PC	FA	BFS
Chemical properties (%)			
CaO	58.85	6.75	32.94
SiO_2_	19.41	63.09	32.37
Al_2_O_3_	5.58	3.74	10
Fe_2_O_3_	3.67	6.11	1.33
MgO	2.12	10.53	9.77
SO_3_	3.16	0.63	0.7
K_2_O	0.69	5.63	0.83
Na_2_O	0.61	–	0.42
TiO_2_	–	1.62	–
SrO	–	0.2	–
P_2_O_5_	–	1.43	–
LOI	6.07	2.6	–
Physical properties			
Specific surface area (cm^2^/g)	4252	2900	4085
Specific gravity	3.17	2.35	2.86

To manufacture the mortars, aggregate with a maximum aggregate size of 2 mm and a fineness modulus of 1.83 was used. Besides, it has a specific gravity of 2.38 and a water absorption of 2.2%. Meanwhile, a polycarboxylate-based superplasticizer (SP) additive with 1.08 g/cm^3^ of density was added to enhance the fluidity (a slump diameter of 25 ± 0.5 cm) of mortars.

### Characterization and preparation of modified WTS

The waste sludge used in the current study is a biological sludge provided from the drying beds of a Waste Water Treatment Plant located in Malatya, Türkiye. This plant has been designed in three phases, with an average project flow rate of 135,000 m^3^/day for 2010; 180,000 m^3^/day for 2020; and 220,000 m^3^/day for 2030, to address approximately 720,000 equivalent population. The treatment process of sedimentation, coagulation, flocculation, and filtration is used in the plant. The pH was between 6 and 9.

The wastes include organic and inorganic compounds in the form of liquid, solid, and gas, whose composition changes as per their chemical, physical, and biological characteristics (Bourgeois et al. 2004). The concentration of heavy metals included in raw WTS was investigated according to the regulation about the use of domestic and urban treatment sludge. The amount of metal and surfactants in raw WTS is due to the irregular discharges of personal cleansing products, household cleaners, and detergents. However, observing Table 2, the heavy metal concentration meets the criteria for the usage of raw waste. The amount of harmful substances can be effectively reduced by the high-temperature calcination of raw WTS, mainly because the heavy metals are transferred to the flue gas and lost (Zhang et al. 2001). Besides, C–S–H and Ca(OH)_2_ generated by the hydration of binders could immobilize heavy metals via chemical bonding as well as physical adsorption in the microstructure (He et al. 2021). Hence, the utilization of MWTS coupled with PC is a sustainable method.

**Table 2:** The heavy metal concentrations in raw WTS

Parameters	WTS (mg/L)	Criteria (mg/L)
Pb	26.78	750
Cd	0.77	10
Cr	65.7	1000
Cu	163.7	1000
Ni	55.16	300
Zn	491.7	2500
Hg	0.16	10

**Fig. 3:**
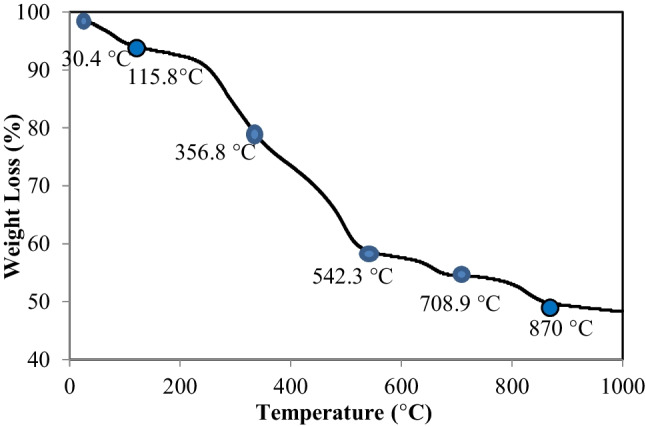
TGA curve of raw WTS

Figure 3 shows the TGA curve obtained from the thermal analysis carried out in a sample of raw WTS. As seen, five different weight losses were found in the raw sample analysis. The first of these weight loss values was between 30.4 and 115.8 °C, and the loss was 4.12%. This weight loss was due to the relative humidity in the structure, which indicates that the sample retains approximately 4.12% moisture. The second mass loss value was 17.81% which occurred between 115.8 and 356.8 °C. This shows the thermal deaggregation of organic groups in the sample. The weight loss between 356.8 and 542.3 °C was around 18.5% which was due to the degradation and carbonization of thermally stable organic groups. The fourth weight loss was seen between 542.3 and 708.9 °C and was around 4.3%. It was caused by the dehydration of surface hydroxyls in inorganic components or the removal of SO_2_ and SO_3_ in some sulfates. The last weight loss occurred between 708.87 and 870 °C. There was a weight loss of approximately 4.88% caused by the removal of CO_2_ in carbonate structures. It was found that the total residue amount of the sample at around 1000 °C was approximately 49.34%. Based on the thermal analysis results, the calcining temperature of raw WTS was planned as 700 °C for 2 h in order not to spoil the carbonates in the structure. The total amount of decomposed matter at 700 °C was found to be 44.5%.

**Fig. 4:**
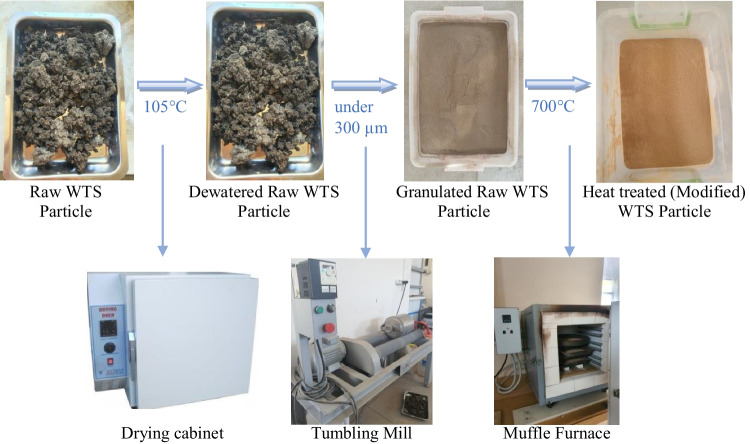
The preparation of modified WTS

**Fig. 5:**
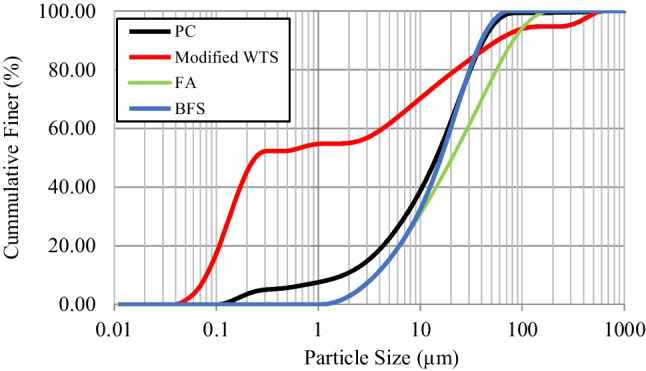
Particle size distribution of binders

**Fig. 6:**
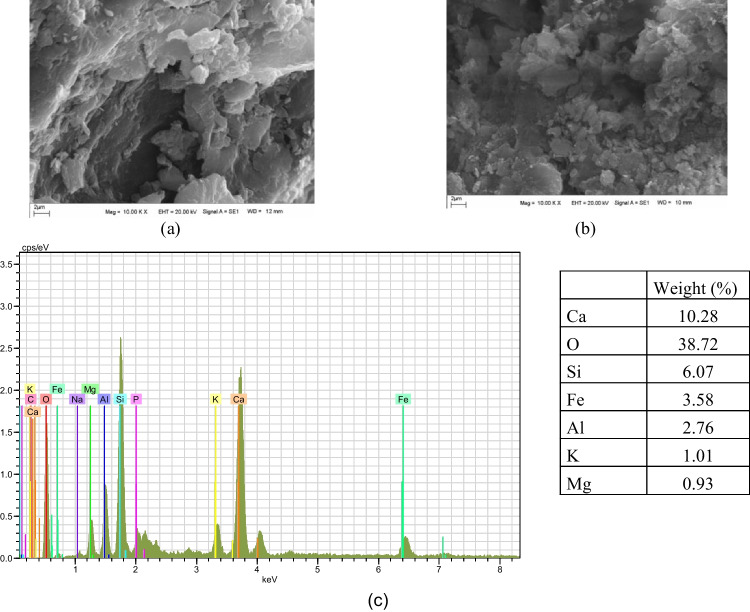
SEM images of (**a**) raw WTS, **b** modified WTS, and EDX result of **c** modified WTS

For the production of modified WTS, at first, the raw waste was dried at 105 °C in a drying cabinet to remove moisture for 24 h. Then, it was crushed under 300 µm using a tumbling mill. After that, the granulated raw WTS was calcined in a muffle furnace at 700 °C for 2 h as was determined through thermogravimetric analysis of the raw WTS. The recycling of WTS and its modification process are presented in Fig. 4. The particle size distribution of the binders (PC, FA, BFS, and MWTS) used in this study is shown in Fig. 5. It was obvious that the average particle size of MWTS was less than those of the other binders. The fineness of calcined WTS was so promising to achieve satisfactory reactivity. Figure 6 shows the SEM images of raw and MWTS. It was seen that their particle morphology was so similar. The chemical composition of MWTS is presented in Table 3 which indicates that MWTS has high content of silica.

**Table 3:** Chemical characteristics of modified WTS

	SiO_2_	Fe_2_O_3_	Al_2_O_3_	CaO	MgO	SO_3_	K_2_O	Na_2_O	MnO	TiO_2_	P_2_O_5_	LOI
(%)	38.49	6.81	11.35	17.93	6.8	0.88	2.46	0.81	0.09	0.77	6.9	6.41

The use of modified (calcined) sludge as a substitute cementitious material was investigated through the examination of the properties of sludge to assess sludge compatibility with PC. The quantitative mineralogical analysis of modified WTS was examined via Fourier transform ınfrared (FTIR) spectrometry and diffraction patterns (XRD).

**Fig. 7:**
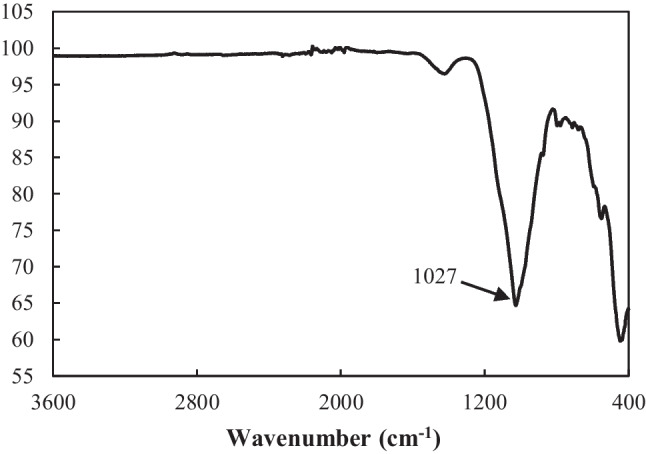
FTIR of modified WTS

Figure 7 shows the transmittance spectra in the infrared region obtained through FTIR spectrometry of MWTS. Si–O–Si stretching band was observed at 1027 cm^−1^ according to the FTIR spectrum which was due to silicate groups in this structure. At 975 cm^−1^ and 850 cm^−1^, Si–O and Al–O peaks were observed, respectively, and it was due to the alumina structure after sintering. At low wavelengths, approximately at 450 cm^−1^, there was a Ca–O peak. Besides, the shoulder at 1100 cm^−1^, Ca–O–Ca peak was observed. The CaCO_3_ structure turned into CaO after the mode. And, at the same time, it was observed that the organic groups disappeared together with the pyrolysis (burning), and an inorganic structure was formed.

**Fig. 8:**
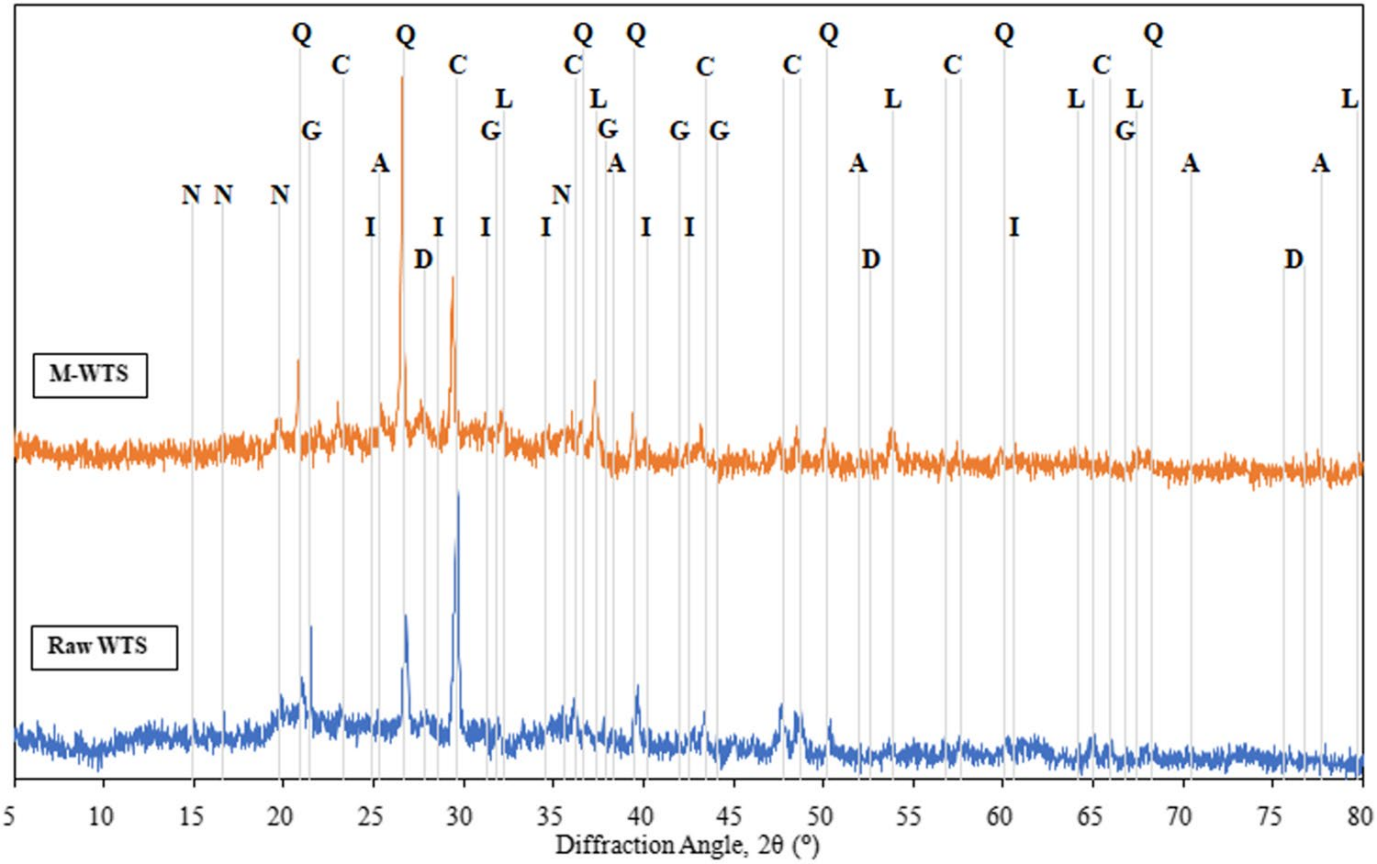
XRD patterns of raw and modified (calcined) WTS; (A, anhydrite (COD 96–901-7280); C, calcite (COD 96–900-1298); D, diopside (COD 96–100-0010); G, gypsum (COD 96–901-3172); I, illite (COD 96–900-9666); L, lime (COD 96–720-0687); N, newberyite (COD 96–900-7633); Q, quartz (COD 96–900-5025))

The powder diffraction pattern of the waste raw sludge identified the presence of calcite (CaCO_3_), quartz (SiO_2_), and gypsum (CaSO_4_.2H_2_O) as major constituents, as shown in Fig. 8. Besides, the main mineral phases detected in waste calcined sludge (MWTS) were quartz, calcite, limestone (CaO), and anhydrite (CaSO_4_). It was observed that the quartz peak intensities increased as expected after the calcination. However, it was seen that calcite did not completely turn into CaO in the waste sludge calcined at 700 °C. This supports the knowledge that the thermal conversion of CaCO_3_ to CaO starts slowly at 600 °C and rapidly decomposes above 750 °C and completely decomposes at 800 °C (Rodriguez-Navarro et al. 2009; Karunadasa et al. 2019). A new mineral, illite, has been identified in calcined sludge. It consists of two silica tetrahedral sheets, and its general formula is K_y_Al_4_(Si_8-y_, Al_y_)O_20_(OH)_4,_ with 1 < *y* < 1.5 (Hongxia et al. 2016). The illite acts as an adsorbent for heavy metal ions by providing an ion exchange reaction with potassium ions trapped in the interlayer spaces and, also, inner-sphere complexes formation through ≡Al − O^−^ and ≡Si − O^−^ groups at the edge (Gu and Evans 2007). The gypsum in the calcined waste sludge turned into anhydrite by removing the H_2_O in its body after calcination. Observing XRD patterns, calcination not only removed the organic materials, but also caused the decomposition of some mineral phases in the waste sludge resulting in the emergence of new mineral phases.

### Mix proportions

In this study, ten mortar mixtures with a water-binder ratio of 0.48 were designed, as given in Table 4. A control mixture was produced with only PC as the binder, while others included binary (MWTS + PC) and ternary (MWTS + FA + PC, MWTS + BFS + PC) blends in which a proportion of PC was substituted with other binders. The binary and ternary blended mortar mixtures were prepared by using MWTS instead of PC at proportions of 10%, 20%, and 30% by weight of cement. Besides, in the ternary blended mixtures, the replacement levels for FA and BFS were 30% by weight of PC. The total binder content was 650 kg/m^3^ for all mixes, and the amount of SP was adjusted to reach a similar flowability (i.e., a mini-slump diameter of 25 ± 0.5 cm).

**Table 4:** Mix proportions of mortars (kg/m^3^)

Mix code	PC	MWTS	FA	BFS	Sand	Water	SP
Control	650	0	0	0	1110	315	20
MWTS10	585	65	0	0	1110	315	23
MWTS20	520	130	0	0	1115	315	26
MWTS30	455	195	0	0	1120	315	26
MWTS10-FA	390	65	195	0	1070	315	23
MWTS20-FA	325	130	195	0	1080	315	24
MWTS30-FA	260	195	195	0	1080	315	24
MWTS10-BFS	390	65	0	195	1105	315	24
MWTS20-BFS	325	130	0	195	1100	315	24
MWTS30-BFS	260	195	0	195	1110	315	24

In the production of mortar mixtures, at first, the sand and 2/3 of the mixing water were mixed during a minute. Then, the binders and SP mixed with the rest of the mixing water were added into the mixer. The mixing was continued for two more minutes. When the target flowability was achieved, the fresh mixtures were poured into specimens. They were demolded after 24 h and cured under laboratory conditions (20 ± 2 °C, 95 ± 3% RH) for 7, 28, and 90 days.

### Testing methods

#### Rheological measurement

Rheology tests were performed using a rate-controlled Brookfield DV-E viscometer with a smooth-walled concentric cylinder at 23 ± 2 °C room temperature. This rotational viscometer was equipped with a Vane geometry spindle. Five hundred milliliters of fresh mortar was placed in a beaker with a height of 100 mm and an inner diameter of 50 mm for the measurement. A constant pre-shearing cycle (100 rpm/2 min) was applied for all mixtures to achieve equilibrium in the matrix response. The plastic viscosity and yield stress of the fresh mortars were determined at rotational speeds varying from 0 to 100 rpm/min and 100 to 0 rpm/min. Then, the flow curve for the ascending and descending legs of the shear rate–shear stress graph was drawn by reading the appointed speeds. The parameters were realized at nine angular speeds (0.5, 1, 2, 2.5, 5, 10, 20, 50, and 100 rpm). Distinct mathematical approaches are present to describe the flow curve of mortar/concrete in the literature (Banfill 2003). In this study, the yield stress (${\tau }_{0})$ and plastic viscosity of fresh mortars were obtained from the flow curve, corresponding to the Bingham model by linear regression (Turk and Demirhan 2017) as given in Eq. 1.

1 $$\tau = {\tau }_{0}+ K.\gamma$$

where $\tau$ is the shear stress (Pa), $\gamma$ is the shear rate (s^−1^), and *K* is the constant. It can also be described as the following:

2 $$T= g+h.N$$

where $g$ is the yield stress (N mm) and $h$ is the plastic viscosity (N mm min). The plastic viscosity defines how easily the mortar flows, while the yield stress defines the shear stress required to initiate the flow of fresh mortar.

#### Strength measurement

Compressive and flexural tensile strength tests were carried out to attain the mechanical properties of the mortar specimens. Three specimens were tested for each test and curing age of 7, 28, and 90. The compressive strength values were measured based on ASTM C39/C39M-20 (2020) using a universal testing machine. The fresh mortars were cast into 40 × 40 × 40 mm cubic molds, and the loading rate of 6 kN/s was used. The test was performed in the ambient condition. Three-point bending test was performed based on ASTM C78 (ASTM C78/C78M-18 2018) using a Shimadzu universal compressive testing machine to determine the flexural strength values. The beam specimens of 40 × 40 × 160 mm dimensions were used, and the clear span was 120 mm. The load was applied at the mid-span of the beam as a single concentrated force. The loading direction was 90 °C rotated from the casting position. Before starting to apply the load, calibration was done, and the loading rate was arranged as 0.5 mm/min. The tests were continued until failure of the mortar samples.

#### Porosity based on MIP

Determination of the pore size distribution and porosity of the mortar samples were measured via mercury ıntrusion porosimetry (MIP), as described in ASTM D4404 (ASTM D 4404 2018). In the test, a sample is put into a chamber surrounded by mercury. A pressure is then gradually applied to the mercury. Thus, the mercury is forced into the pores of the sample. This pressure ($$P)$$ is called the Washburn equation as seen below (Li et al. 2010):

3 $$d=\frac{-4\gamma {\text{cos}}\phi }{P}$$

where *d* is the apparent pore diameter, $\gamma$ is the surface tension of the mercury, and $\phi$ is the contact angle between the pore wall and mercury. Before testing, the inner core of the cured samples was cut, and 3-g pieces, which were approximately 3–5 mm, were taken. They were dried at 105 °C for a day to remove the water, and then, they were immersed in ethanol for 24 h to stop the hydration process.

#### Microstructural and mineralogical characterization

The produced and cured specimens were carefully cut into 10-mm pieces, and then, in order to stop the hydration reactions by removing free water from the samples, they were immersed in ethanol. After that, small mortar particles were ground until the maximum particle size of the powder was less than 45 µm. The mineralogical characteristics of the powder were identified by XRD patterns which are examined through a Rigaku Rint 2000 X-ray Diffractometer between 2 and 80°/min with a scanning rate of 2°/min. CuKα radiation with a wavelength of 1.5405 Å at a voltage of 30 kV was used. Besides, for FTIR analysis, a few milligrams of powdered samples were dispersed in approximately 80 mg of spectral-grade potassium bromide (KBr) and pressed into pellets at a pressure of approximately 10 t/cm^2^. The measurements were conducted using the Perkin Elmer, Spectrum One system that was operated in the absorbance mode. The spectra are normally obtained using 4 cm^−1^ resolution yielding IR traces between the wavenumber range of 400 and 4000 cm^−1^. All data were corrected for pure KBr spectrum.

In addition, SEM was used to identify the surface structure of the mortar samples. To analyze the morphology of the hydrated composite, the fractured pieces taken from the samples subjected to a compressive strength test were used. These pieces were dried at 105 °C for 24 h, and then, the analyses were carried out using SEM/EDX system (Leo, EVO-40 VPX, UK) at 20 kV acceleration voltage. Before SEM examination, in order to avoid charging effects, the samples were coated with gold thin.

## Results and discussion

### Rheological performance

**Fig. 9:**
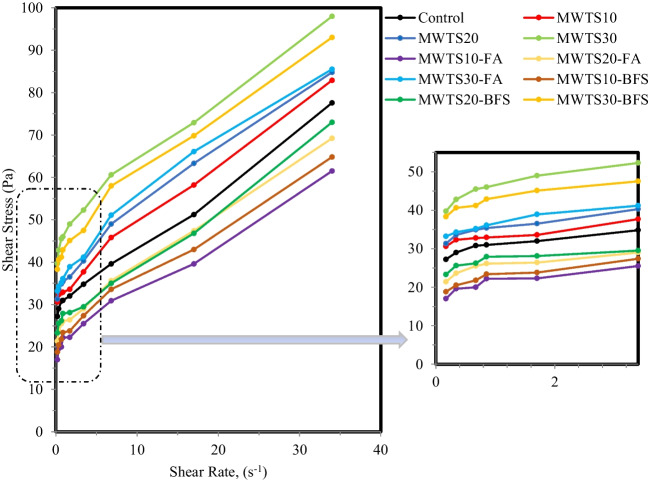
Flow curves of rheology tests of mortars

**Fig. 10:**
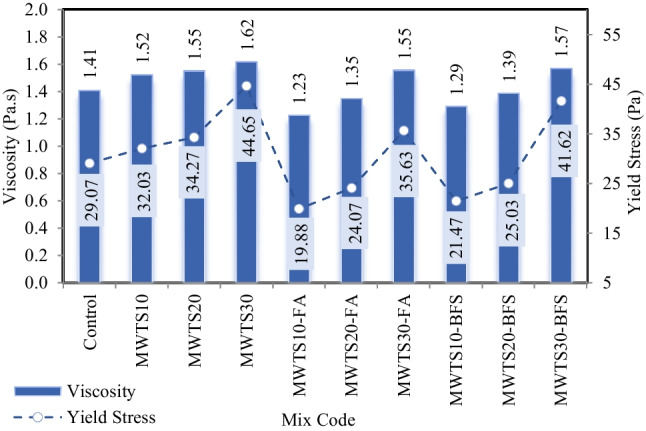
Impact of waste products on the viscosity and yield stress of fresh mortars

Rheological properties are more objective and accurate than traditional workability testing methods to evaluate the deformation and flow ability of cementitious composites under external shear stress. In order to analyze the rheological parameters of each fresh cement mortar, the data obtained in the down curve of the data recording process in the rheology measurement process was utilized due to its repeatability and reliability (Kwan et al. 2010). Regression analysis was performed to obtain the best flow curve, and based on the results, the modified Bingham model best fitted the data, unlike other rheological models. The flow curves of each mortar are displayed in Fig. 9, and based on the Bingham equation (Eq. 1), the yield stress and plastic viscosity parameters are obtained and plotted in Fig. 10.

Yield stress is the minimum shear stress required for the mortar to flow from the static state. It is affected by the network structures within the cement composites. The network structures are formed by the zeta potential, electronic repulsion, and van de Waals attraction forces between the cement particles and the flocculation of cement hydration (Cao et al. 2018). Besides, plastic viscosity is an indicator of the resistance of the mortar against flowing. The effects of different mineral admixtures on the rheology of fresh mortar can be determined in terms of their particle size distribution, material and surface texture, shape, concentration, morphology, etc. (Kwan and Fung 2012).

Observing Fig. 9, the binary blended mortars with MWTS possessed higher shear stress values relative to the control mixture. The yield stress values increased with the increasing dosages of MWTS as a replacement of cement in a binary system ranging from 32.03 Pa (MWTS10) to 44.65 Pa (MWTS30), and also, it was seen that the incorporation of MWTS increased both the yield stress and viscosity of the control mixture. It can be explained by the fact that the partial substitution of cement with MWTS, which has a finer particle size distribution and therefore a higher specific surface area, reduced the distance between particles. Therefore, it can increase the friction and probable collision between the grain particles, resulting in increasing yield stress. Similar behavior was also reported by other researchers (Perrot et al. 2012; de Matos et al. 2021; Ruviaro et al. 2021) who observed an increase in the yield stress with the utilization of wastes into cement matrices. On the other hand, viscosity was not significantly affected for the use of MWTS up to 20% (1.52 Pa.s and 1.55 Pa.s), while 30% replacement of cement with MWTS increased the viscosity by 14.87% relative to the control mixture. Furthermore, it can be said that the utilization of 30% MWTS as a replacement of cement by weight caused a higher filler effect due to having much more MWTS particles, thus causing the matrix composite to change from the mobile phase to the stationary phase, resulting in higher yield stress and viscosity for fresh mortar. Consequently, the presence of higher MWTS dosage required high energy to attain good flowability relative to the control mixture. Mohsen et al. (2022) used untreated lead sludge as a replacement for cement and also found that due to the high surface area of sludge, the interlocking between cement grains increased, thus preventing the flow of particles and decreasing the viscosity. Besides, in the study conducted by Ruviaro et al. (2021), it was determined that substituting up to 20% WTS with cement caused small increases in yield stress. However, marginal changes were observed in the plastic viscosity up to 40% incorporation. Spat Ruviaro et al. (2023) also used eggshell filler and WTS at the replacement of 10%, 15%, 20%, and 30% with cement. They found a progressive increase in the yield stress as the increase in WTS content. It was concluded that 45% replacement of cement with WTS dominated the rheological behavior due to its high surface area.

Regarding ternary blended mortars, the incorporation of 10% and 20% MWTS with FA/BFS shifted the flow curves to lower yield stress values relative to the control mixture, which may be a sign of improved rheological behavior of the fresh mortar. The reduction in yield stress and viscosity was more pronounced for the ternary blended mortars with FA compared to mortars with BFS. For example, the ternary use of 10% MWTS and FA had the lowest viscosity and yield stress values with a reduction of 12.92% and 31.63%, respectively, relative to the control mixture, while these reduction values were 8.36% and 26.16% for MWTS10-BFS mixture, respectively. The higher enhancement in the rheological properties of mixtures with FA can be attributed to the smooth, spherical shape of FA particles. Thus, it provides ball-bearing effects which can decrease the friction force between the cement particles (Kutchko and Kim 2006; Liu et al. 2021). Besides, the particle size distribution of FA was coarser than the other waste products used in this study (see Fig. 5) which reduces the water demand. In addition to these, the improvement of the rheological parameters by the addition of BFS into the mortar with MWTS can be explained by its wide particle size distribution relative to cement. It increases the free water and facilitates particle packing. Similar findings were also proven by other researchers (Park et al. 2005; Grzeszczyk and Janowska-Renkas 2012; Adjoudj et al. 2014). On the other hand, for all fresh mortar mixtures, the addition of 30% MWTS as a binary/ternary blend led to a significant increase in both viscosity and yield stress. For example, the viscosity of MWTS30, MWTS30-FA, and MWTS30-BFS was 14.68%, 10.24%, and 11.23% higher than that of the control mixture, while these values were 53.56%, 22.56%, and 43.16%, for yield stress, respectively. It can be explained by the high amount of irregular MWTS particles which increase the internal friction during flow. Consequently, it was observed that the drawbacks of the use of MWTS as a replacement for cement, in terms of rheological properties, were suppressed by the utilization of FA and BFS as a ternary blend. In fact, the utilization of an appropriate dosage of MWTS with FA/BFS improved the rheological parameters of mortar relative to the control mixture with only PC. From this result, it can be emphasized that the utilization of high content of waste by-products in cement-based materials can be a potential application.

### Mechanical properties

#### Compressive strength

**Fig. 11:**
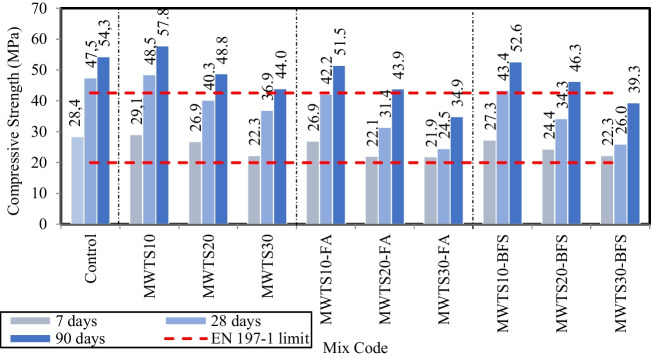
Compressive strength of mortar samples having various waste products

The compressive strength values of mortar samples having various waste product content for 7, 28, and 90 curing days are shown in Fig. 11. First of all, it is worth noting to say that all compressive strength values at 7 days meet the requirements (≥ 20 MPa for 7-day curing age) of EN 197–1 standard (BS-EN197-1: 2011) for 42.5 N resistance class cement. However, among all 28-day samples, only the binary usage of 10% MWTS and its ternary blends with FA/BFS satisfied the limitation (≥ 42.5 MPa for 28-day curing age) of EN 197–1 standard (BS-EN197-1: 2011).

The use of 10% MWTS as a replacement of cement by weight caused a slight (2.29%) increase in the compressive strength values relative to the mortar with only PC as a binder at an early age, which could be due to the filler effect of MWTS. As the age of curing prolonged, the MWTS went through a secondary hydration reaction with PC hydration products, resulting in improving the compressive strength of the mortar. It was found that the MWTS10 sample had 6.4% higher compressive strength than that of the control sample at 90 days. This can be attributed to the smaller particle size of MWTS and its pozzolanic activity. The results pointed out that MWTS had a tendency to promote a filler effect and react with Ca(OH)_2_ present in the hydrated cement, which improves the particle packing. Therefore, the mechanical and physical behavior of the material produced with MWTS was improved. On the other hand, the replacement of a higher amount (20%, 30%) of MWTS with PC reduced the compressive strength values. That is, MWTS30 had 19.0% lower 90-day compressive strength than that of the control sample, which can be due to the low reactivity of MWTS. This result is consistent with the recent studies (He et al. 2021; Duan et al. 2022).

Observing Fig. 11, all ternary blended mortars exhibited lower strengths compared to the control sample for all ages. Besides, the addition of FA or BFS to the mortars having MWTS decreased the compressive strength values relative to the mortar samples blended as binary with MWTS. At early ages (7 days), for all proportions of MWTS, BFS provided higher compressive strength than FA. That is, the ternary use of 10% MWTS and FA caused a 5.1% reduction, while a decrease in 7-day compressive strength was 3.8% for the ternary blends of MWTS10-BFS. This may be because FA is not a hydraulic material. It has insufficient CaO content with regard to BFS, so the hydration process cannot occur on its own. In order to increase the binding property of FA, an activator such as Ca(OH)_2_ should be used to increase the hydration efficiency of cement or to initiate the hydration reactions in cement (Teixeira et al. 2022). Although the CaO content of MWTS (see Table 3) was higher than that of FA, it was seen that its contribution to the compressive strength gain became also insufficient, such that the increase in the substitution rate of MWTS with cement caused a further reduction in compressive strength which could be due to lower cement content. For example, the ternary blends of MWTS20-FA and MWTS30-FA showed a decrease of 21.3% and 23% in early strength compared to the control samples, respectively. Moreover, the ternary use of MWTS and BFS resulted in higher early compressive strength due to its higher CaO content (Taylor et al. 2010; De Weerdt et al. 2011). On the other hand, MWTS10-FA and MWTS10-BFS had similar 90-day compressive strengths, with values of 51.5 MPa and 52.6 MPa, respectively, which were 5.16% and 3.13% lower than that of the control sample. This result can be explained by having higher SiO_2_ content of FA relative to BFS. It can lead to an enhancement in the late compressive strength as a result of the pozzolanic reaction between Ca(OH)_2_ and SiO_2_. Besides, it was also thought that the utilization of MWTS with FA as a ternary blend also contributed to the development in the 90-day compressive strength due to the presence of higher SiO_2_ content of MWTS (see Table 3). On the other hand, the decreasing trend in late strength became obvious as the MWTS ratio increased. That is, MWTS20-FA and MWTS30-FA samples exhibited 19.1% and 35.7% lower 90-day compressive strength relative to control samples, respectively, while these values were 14.7% and 27.6% for MWTS20-BFS and MWTS30-BFS, respectively. It showed that the ternary use of 20% and 30% MWTS with BFS had higher late strength compared to those of the samples having MWTS and FA. This was due to the reason that the small particles of cement and MWTS are hydraulically absorbed by BFS via chemical reaction (Cong et al. 2020; Fan et al. 2020). BFS includes a high content of CaO and performs as a hydraulic material that hardens upon the addition of water through a chemical process (Siddique and Kadri 2011). After activation of hydraulic activity, BFS reacts with free Ca(OH)_2_ and moisture from the hydration products to produce more C–S–H, which improves strength gain (Islam et al. 1970). Besides, it can create stronger bonding in the matrix by filling the voids (De Belie et al. 2018) with the contribution of MWTS which has a much finer grain size relative to other binders. However, observing Fig. 10, as the content of waste products increased, the compressive strength values decreased significantly due to the lower cement content.

#### Flexural strength

**Fig. 12:**
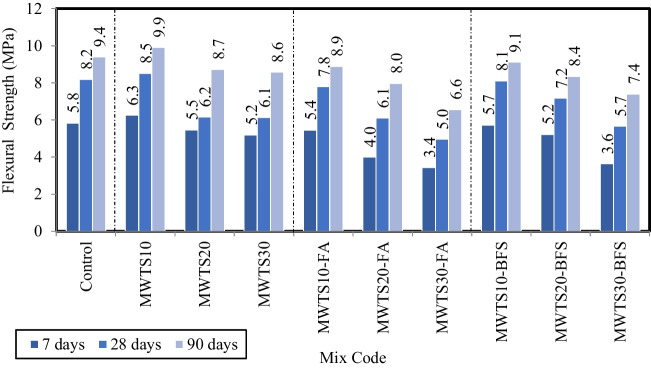
Flexural strength of mortar samples having various waste products

Figure 12 indicates the flexural strength values of mortar samples having various waste product proportions for 7, 28, and 90 curing days. The flexural strength values of the samples slightly increased with the addition of 10% MWTS as a replacement of cement by weight. That is, the flexural strength of MWTS was 5.4% higher than that of the control sample. The filler effect and pozzolanic activity resulting from the finer particle size distribution of MWTS contributed to the flexural strength of the samples, as in the case of compressive strength. This enhancement in strength can also be explained by the higher amount of reactive silica produced by the use of MWTS. Thus, the pozzolanic reaction between minerals in the WTS and the cement hydrates can be improved, which may cause a contribution to the strength gain (Teixeira et al. 2011; Huang and Wang 2013). However, as the substitution rate increased from 10 to 20% and 30%, the flexural strength values significantly decreased. The use of 20% and 30% MWTS as a replacement for PC reduced the 90-day flexural strengths by 7.2% and 8.8% with regard to a control sample, respectively. It can be explained by the reduced availability of cement for hydration and thus the delay in the development of hydration products such as Ca(OH)_2_ and C–S–H (Fang et al. 2019).

Considering Fig. 12, the ternary use of waste products caused a reduction in the flexural strength relative to the control sample, and also, the incorporation of BFS or FA with MWTS reduced the strengths of binary blended mortars with MWTS for all curing days. Besides, in the ternary blended systems, the use of MWTS with BFS resulted in better results in terms of flexural strength as in the case of compressive strength. Especially, the highest flexural strength values were obtained from MWTS10-BFS with 5.7 MPa, 8.1 MPa, and 9.1 MPa for 7, 28, and 90 days, while they were obtained as 5.4 MPa, 7.8 MPa, and 8.9 MPa for MWTS10-FA, respectively. It may be attributed to the fact that BFS and MWTS had finer particle size distribution relative to FA (see Table 3), which provides a greater surface contact area for the reaction to occur (Desta and Jun 2018). Moreover, the surface texture and shape of BFS had a positive impact on flexural strength (Johnston 1998). On the other hand, as the MWTS content increased, a significant reduction in flexural strength was observed for all ternary blended mortars due to having lower cement content at all curing days. Since the development of pozzolanic activity is dependent on Ca(OH)_2_ obtained from the hydration of cement, some of the mineral additives in excess remain inactive due to the decreased cement content, and thus, the strength contribution ends. This result is consistent with the studies in the literature (Saha 2018; Turk and Kina 2018; Ozturk et al. 2020; Yun et al. 2020; Turk et al. 2023). Consequently, the strength test results indicated that the utilization of an appropriate amount (10%) of MWTS with 30% FA or 30% BFS as ternary blend into mortar was possible for their potential application in cement-based materials to minimize the cement dosage and increase the usage of waste products.

#### Cement ıntensity

The cement intensity (CI) values of the designed mortars were calculated using the binder ıntensity calculation proposed by Damineli et al. (2010). It measures the amount of cement necessary in 1 m^3^ to obtain a compressive strength of 1 MPa. The value of CI was evaluated using the 90-day compressive strength values of the mortars due to having pozzolanic reactions.

**Fig. 13:**
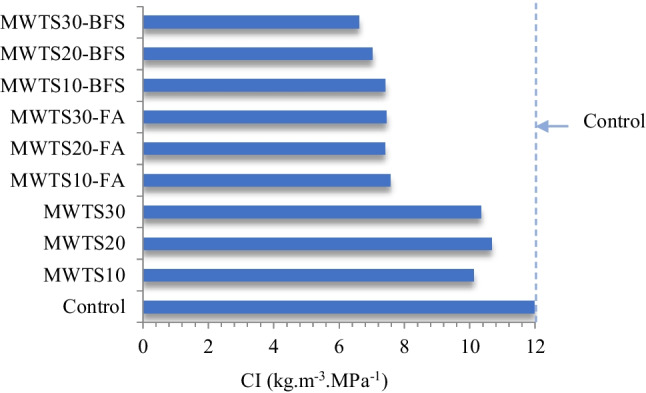
Cement Intensity values of the mortars at 90 days

Observing Fig. 13, the control sample had a CI value of 11.97 kg m^3^. Besides, regardless of the ratio, the replacement of MWTS with cement showed a similar effect, such that the value of CI was found as in the range of 10.12 kg m^3^ and 10.66 kg m^3^. However, after the addition of FA and BFS, the CI values varied between 6.62 and 7.45 kg m^3^, and MWTS30-BFS had the lowest CI value at 6.62 kg m^3^. In terms of sustainability, an increase in the waste product content caused a lower need for cement to achieve 1 MPa of compressive strength. From this study, the possibility of using PC contents lower than that used in the control sample to produce mortars with good compressive strength was proven. It can be emphasized that the utilization of MWTS with FA or BFS as ternary blend into mortar sample as a replacement of PC had a significant role to obtain sustainable cementitious material due to having lower CI values relative to the control sample indicating that less amount of cement was needed to achieve unit strength. It is also observed that the ternary blend of MWTS and BFS presented the best results due to their finer particle size distribution and higher consuming Ca(OH)_2_, resulting in better pore refinement, verified in the MIP and microstructural analysis (XRD), as discussed below. In the aspect of having slightly lower cement index values and a lower rate of decrease in strength relative to the control sample, it can be emphasized that the use of 10% MWTS with BFS as a ternary blend was possible to produce sustainable cement-based materials.

The CI values found in this study were consistent with some researches in the literature. Medeiros et al. (2021) used porcelain polishing residue and scheelite residue as a partial substitution for cement and sand, respectively. The concrete designed with 30% replacement of PC with porcelain polishing residue had the lowest CI value with 7.89 kg m^3^. In the study by Matos et al. (2018), the average of the CI values of the self-compacting concrete blended with porcelain polishing waste with the replacement of 10, 20, and 30% cement was found as 7.9 kg m^3^. Diniz et al. (2022) used sugar cane bagasse ash, rice husk ash, and metakaolin as mineral admixtures by cement replacement up to 60%. It was found that the metakaolin and rice husk ash combination gave the best result due to having a lower CI value. Donmez et al. (2023) substituted cement by BFS, FA, and limestone as binary, ternary, and quaternary with different proportions to produce fiber-reinforced self-compacting concrete. The lowest CI value was found as 4.60 kg m^3^ for the hybrid fiber-reinforced concrete blended as ternary with FA and BFS.

### Porosity

Porosimetry is a significant parameter in terms of durability evaluation since it can assess the pore network of cementitious materials. The pore structure of the mortars was analyzed by MIP, and the results were expressed by the pore size distribution curves in Fig. 15. The gel pores are considered as less than 10 nm, and capillary pores are between 10 and 2000 nm (Naqi et al. 2020). The pore volume greater than 50 nm is considered to have an important influence on strength (Naqi et al. 2020) and permeability, while the smaller pores affect the drying shrinkage (Kumar Mehta and Monteiro 2014).

**Fig. 14:**
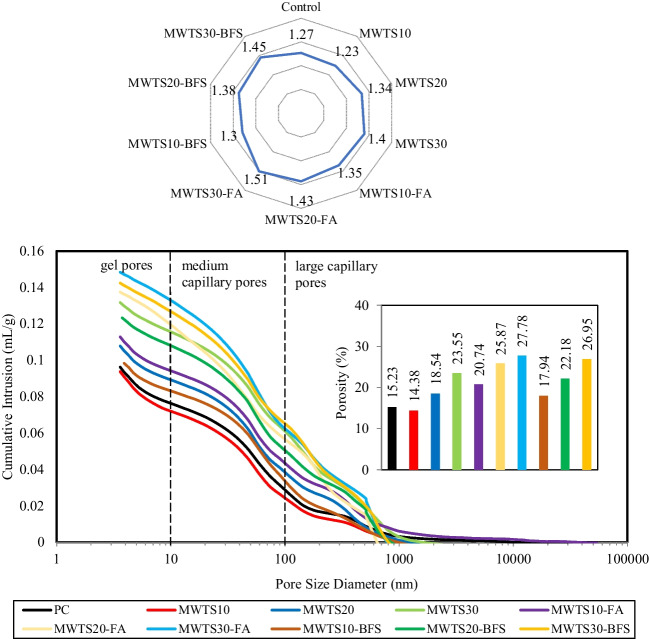
Pore structures of the mortar samples

As visible in Fig. 14, the pore size distribution curves of the control sample and MWTS were so similar, while MWTS10 had the lowest pore volume with the lowest porosity value of 14.38%. It showed that the incorporation of 10% MWTS with cement by weight resulted in reduced pore diameter and lower porosity. Considering Fig. 14, an increment in gel pores is so obvious which signifies an additional formation of C–S–H from the pozzolanic reaction. This increase in C–S–H content has contribution to the strength improvement as seen in Fig. 11. However, more amount of MWTS (20% and 30%) caused a higher pore volume in binary blended mortar samples. This indicated that the use of 10% MWTS as a partial substitution of cement enhanced the compactness of the structure due to its filler effect caused by the fine particles. However, the pozzolanic reactivity of MWTS was not pronounced when a higher amount of MWTS was added, thereby increasing the pore volume. These observations are confirmed by the average diameters, as shown in Table 5.

**Table 5:** Porosity and average pore diameter of mortars measured by MIP

	Control	MWTS 10	MWTS 20	MWTS 30	MWTS 10-FA	MWTS 20-FA	MWTS 30-FA	MWTS 10-BFS	MWTS 20-BFS	MWTS 30-BFS
Porosity (%)	15.23	14.38	18.54	23.55	20.74	25.87	27.78	17.94	22.18	26.95
Average Diameter (µm)	0.0193	0.0180	0.0216	0.0287	0.0254	0.0294	0.0323	0.0222	0.0277	0.0321

Observing Fig. 14, all ternary blended mortars attained a higher volume of pores relative to the control sample, but their average pore diameter values were not higher than 50 nm. Besides, the addition of FA/BFS into binary blended MWTS samples increased the volume of large capillary pores resulting in higher porosity. However, the porosity of the mortars having BFS and MWTS as a ternary blend was lower relative to the ones having both FA and MWTS. The higher porosity of the ternary blended samples with MWTS and FA can be explained by having lower reactivity and amorphicity of FA relative to BFS (Nedunuri et al. 2020). Namely, MWTS10-BFS had the lowest porosity and average pore diameter with 17.94% and 0.0222 µm, respectively, among all ternary blended mortar which is in agreement with the strength test results. The incorporation of BFS with MWTS caused the formation of hydration products from the pozzolanic/hydraulic reaction, and these products filled the large capillary pores resulting in a lower volume of medium capillary pores. This causes a densification of microstructure which enhances the strength of the mortar. On the other hand, Fig. 14 indicated that the mortars having 30% MWTS in ternary systems caused a lower fraction of gel pores which reveals the presence of a large amount of unreacted particles.

### Correlations between strength, density, and porosity

**Fig. 15:**
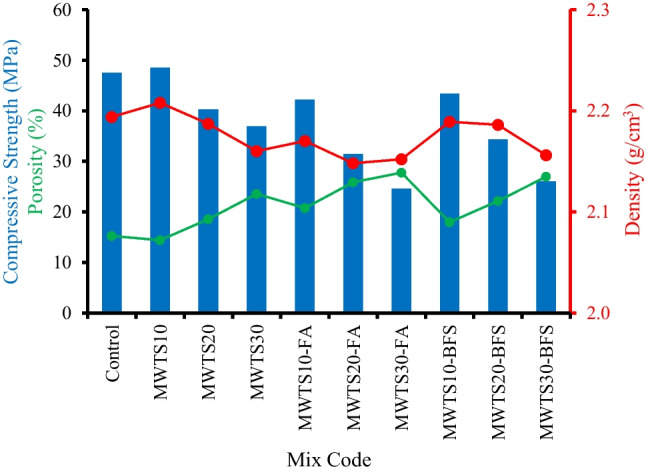
Relationship between strength, density, and porosity

The relationship between the compressive strength, porosity, and density of the samples in the saturated surface dry state is reported in Fig. 15. The increase in the porosity ratio decreased the compressive strength gain of the samples as expected. On the other hand, the density values of all samples were so close to each other, but it is worth to say that there was a linear relationship between the strength and density which may be due to the microstructure densification of the mortars (Rodrigue Kaze et al. 2021). The sample of MWTS10 had the highest compressive strength (48.5 MPa) and density (2.209 g/cm^3^) values and the lowest porosity (14.38%). In ternary blended systems, the incorporation of 10% MWTS with FA or BFS had higher strength and density values and lower porosity which can be due to having a denser structure. The addition of 20% and 30% MWTS as binary and ternary blend decreased the strength and density values by generating pores in the structures as proven in the SEM images in the “Mineralogy of hydrated products” section.

**Fig. 16:**
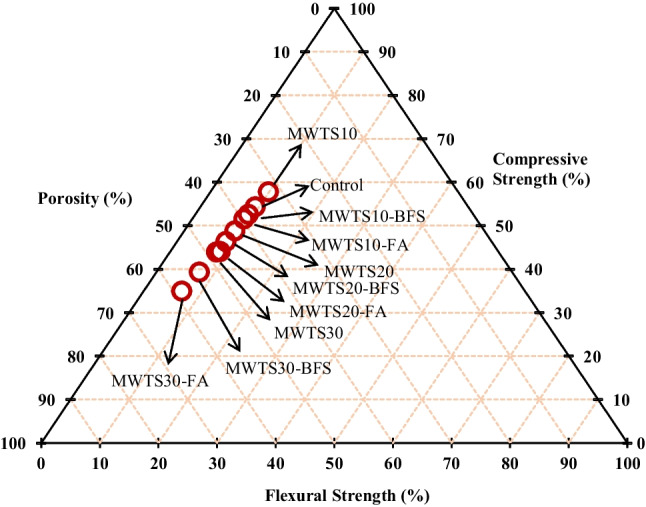
Ternary diagram of the results in hardened state

The ternary diagram of the porosity, compressive strength, and flexural strength results are presented in Fig. 16 to determine the best waste binder content. Binary blended mortar with 10% MWTS presented the best results with the highest compressive and flexural strengths and lowest porosity values. Besides, among the ternary blended systems, the incorporation of 10% MWTS with FA/BFS caused higher strengths and lower porosity. This illustration proved that the optimum percentage of MWTS incorporation was 10 wt% for both binary and ternary blended systems. On the other hand, due to having lower compression and flexural strengths and higher porosity values, the use of 30% MWTS as binary and ternary presented the worst results.

### Mineralogy of hydrated products

First of all, it should be understood that SiO_2_ in cement samples and sand, which will form the main polymeric skeleton in concrete, contains functional groups with O–Si–O structural formula and Si = O double bond. After the mortar is formed and setting (polymerization) begins, SiO_2_ undergoes a structural change and transforms into tetrahedral silicon tetroxide (silicate) form, forming the actual mesh skeleton of the concrete. However, in the center of the lattice, strong octahedral Si–O–Metal bonds begin to form with hydrogen bonds after including hydrated water. These mentioned metals are the ones such as Al, Ca, and Mg contained in metal oxides (Al_2_O_3_, MgO, and CaO) found in cement samples. Therefore, the bonds that will form in the lattice are such as Si–O–Si, Si–O–Al, Si–O–Mg, and Si–O–Ca. When compounds such as SiO_2_, which initially have double bonds, form Si–O sigma bonds in silicate form in the concrete lattice, the peaks in the infrared (IR) spectrum shift to a lower area, that is, the wave number. For example, while sharp peaks are observed in SiO_2_ at 1093, 796, and 471 cm^−1^ in response to stretching vibrations, in the silicate form [SiO_4_]^4−^, overlapping peaks are observed as broader bands between 1000–800 cm^−1^ and 600–500 cm^−1^ (Hu et al. 2012).

**Fig. 17:**
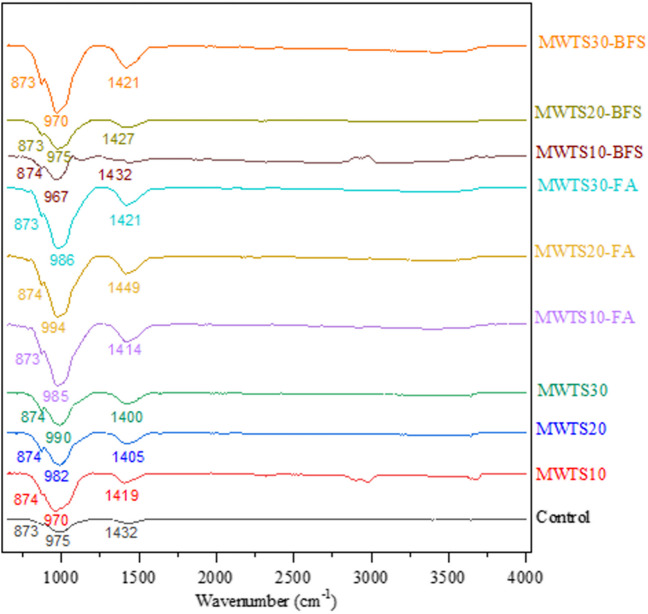
FTIR spectrum of the mortars at 28 days

The FTIR spectrums of the mortars blended by waste products were examined as can be seen in Fig. 17. The bands at 873–874 cm^−1^ were assigned to OH bending in Si–OH groups (Taylor 1990). Observing Fig. 7, a sharp peak with 1027 cm^−1^ was observed in the FTIR spectrum of calcined WTS due to the symmetric and asymmetric stretching vibrations of the SiO_2_ structure. It was observed that in the mortar samples formed by the addition of MWTS with other waste products in certain proportions, this peak (1027 cm^−1^) turned into bands consisting of many more broad and overlapping peaks in Si–O–Si or Si–O–Ca, Si–O–Mg, and Si–O–Al bond lattices with octahedral central structure. That is, the peak wavenumbers were found as 975, 970, 982, 990, 985, 994, 986, 967, 975, and 970 cm^−1^ for control, MWTS10, MWTS20, MWTS30, MWTS10-FA, MWTS20-FA, MWTS30-FA, MWTS10-BFS, MWTS20-BFS, and MWTS30-BFS, respectively. This is the proof that the concrete mesh has formed. Besides, the band at around 3100 cm^−1^ was observed in the FTIR spectrum of MWTS10 and MWTS10-BFS, indicating the O–H vibrations of the H_2_O molecule. The wavelengths between 1400 and 1570 cm^−1^ reflect the low crystalline CaCO_3_ or unreacted calcite (Liu et al. 2020). The peaks at 1400–1449 cm^−1^ seen in all mortar samples are evidence of the presence of Ca–O bond in the structure (Aslan and Gürocak 2022). Additionally, all these peaks are wide which also indicates the existence of having amorphous CaCO_3_ (Andersen and Brečević 1991). These explanations agree with the information in literature (Park et al. 2002; Horgnies et al. 2013; Vaičiukynienė et al. 2013; Kiefer et al. 2018; Duan et al. 2022).

The XRD patterns of hydration products of mortar specimens having various waste products are shown in Fig. 17. The major crystalline phases identified were alite (unreacted C_3_S), belite (unreacted C_2_S), portlandite, and quartz. It was seen that the incorporation of different waste products did not change the main hydration products. The characteristic peaks of Ca(OH)_2_ include 2θ = 17.94°, 28.3°, 33.95°, 46.9°, and 50.7°. The characteristic peaks of quartz include 2θ = 20.6°, 26.5°, 42.3°, and 49.9°. The XRD patterns provide a better comprehension of the pozzolanic activity according to the peak intensities of Ca(OH)_2_ of a material. The control sample had the highest peak intensity of Ca(OH)_2_ generated from the hydration of C_3_S and C_2_S. The utilization of waste products lowered the peak intensity of Ca(OH)_2_ due to the consumption of Ca(OH)_2_ which also proves the pozzolanic activity. However, the peaks of hydration products could be seen in all mortar samples, proving the hydration of cement and justifying the attained strength. The other researchers (Chen and Poon 2017; Hsu et al. 2018; Siddique et al. 2021) also reported similar hydration products.

**Fig. 18:**
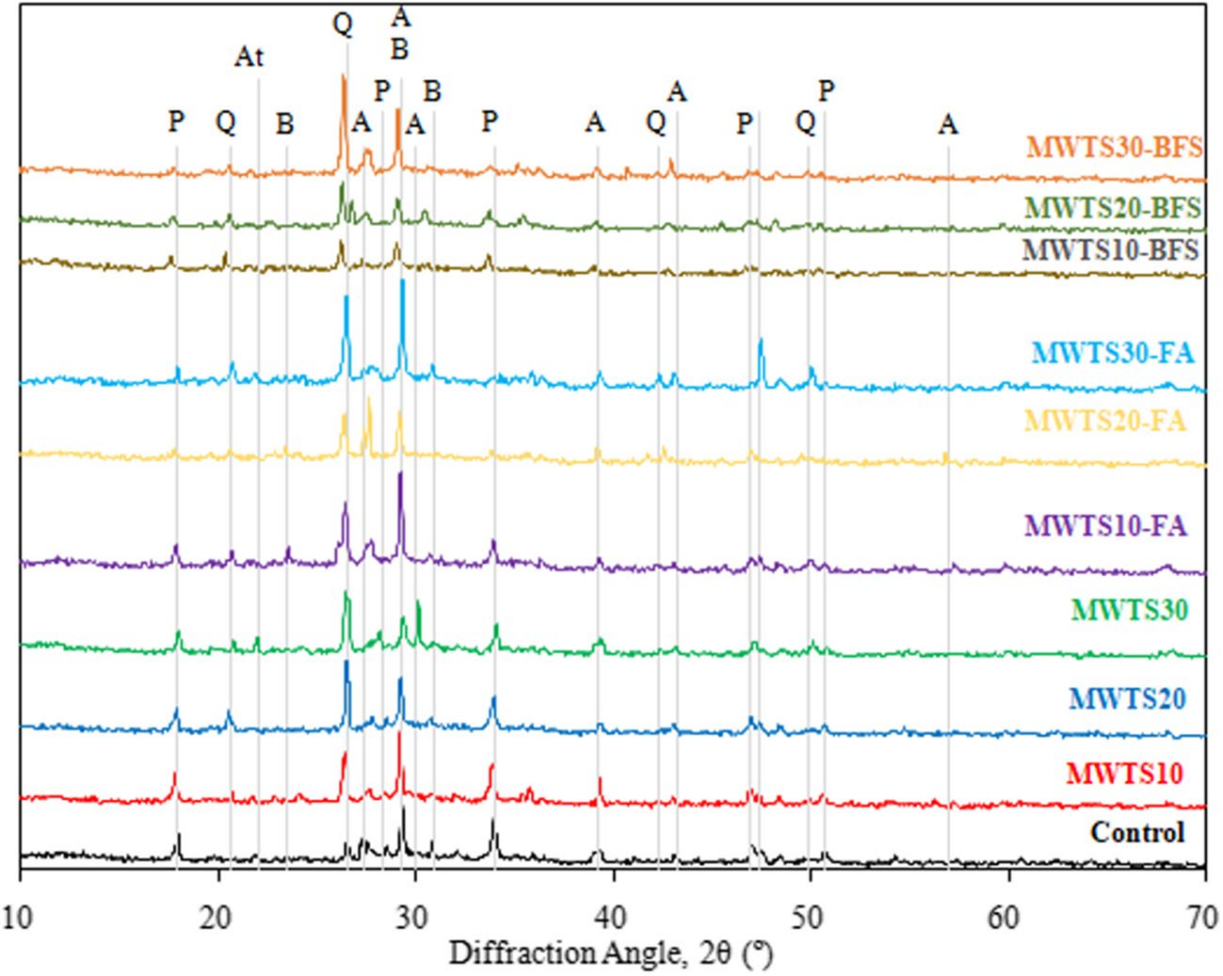
XRD patterns of mortars at 28 days; (A, alite (COD 96–154-0705); At, alite–triclinic (COD 96–901-6126); B, belite (COD 96–231-0676); P, portlandite (COD 96–900-0114); Q:Quartz (COD 96–900-5021))

Considering Fig. 18, the intensity of the peaks of unhydrated alite and belite was higher which was due to the absence of pozzolanic activity. Especially, in the ternary blended mortars having FA, due to its slower reaction rate (Moghaddam et al. 2019), the peaks of belite were observed with higher intensity. At later ages, C_3_S and C_2_S can reduce when the pozzolanic reaction consumes Ca(OH)_2_ to form tobermorite (C–S–H) gels which contribute to the later strength. The characteristic peaks of Ca(OH)_2_ include 2θ = 17.94° and 2θ = 33.95° and were more obvious to compare the intensities as seen in Fig. 17. For the ternary blends of MWTS and BFS, the peaks of portlandite, alite, and belite were lower than that of the ternary blends of MWTS and FA, in general. Namely, at 2θ = 17.94°, the Ca(OH)_2_ peak intensities of ternary blended mortars with MWTS and BFS were 58–60% lower than the control sample, while it was less in the range of 30–45% for the mortars with MWTS and FA. At 2θ = 33.95°, 59–62% lower peak intensities were obtained for the mortars with MWTS and BFS, and it was 30–56% for the ones blended with MWTS and FA. It shows that more Ca(OH)_2_ was consumed by the incorporation of BFS, MWTS, and cement. A probable reason for the reduction in Ca(OH)_2_ could be due to the increased reactivity of MWTS and BFS, which have finer particle size distribution relative to FA, and thus, they have higher surface area for pozzolanic reactivity leading to an increase in nucleation site. However, there were still large amounts of unreacted C_3_S and C_2_S for all mortar samples which shows that the hydration process will continue in the following days. In addition, the characteristic peak intensity of quartz is obvious especially at 2θ = 26.5°. Observing Fig. 18, the SiO_2_ peaks were prominent for all samples, especially the ones having a higher amount of MWTS. This proves that the MWTS includes amorphous SiO_2_ and SiO_2_ crystals. The amorphous SiO_2_ can react with Ca(OH)_2_ to form C–S–H gel, thereby reducing the portlandite peak. The reason for having a higher intensity of SiO_2_ for the specimens having a higher percentage of MWTS can be attributed to the fact that the amorphous SiO_2_ could not react with Ca(OH)_2_ due to its insufficiency caused by the lower cement content. Even C_3_S and C_2_S could not also be consumed by Ca(OH)_2_. This finding is also evidence of lower strength gain for the samples blended with a higher dosage of MWTS. On the other hand, the peak intensity of quartz decreased significantly in the mortars blended with both MWTS and BFS due to the higher alkaline activation caused by BFS (Kamath et al. 2021).

### SEM analysis

**Fig. 19:**
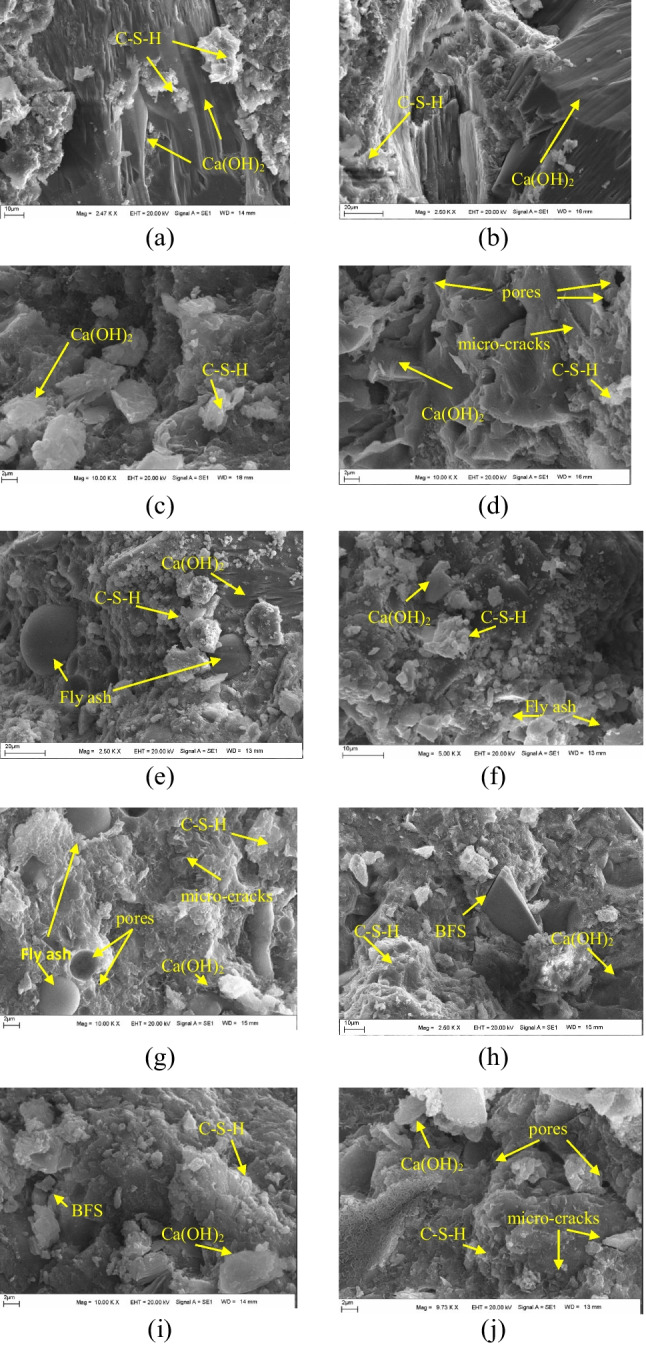
SEM morphologies of the mortar specimens at 28 days of (**a**) control, **b** MWTS10, **c** MWTS20, **d** MWTS30, **e** MWTS10-FA, **f** MWTS20-FA, **g** MWTS30-FA, **h** MWTS10-BFS, **i** MWTS20-BFS, and **j** MWTS30-BFS

Scanning electron microscopy analysis was used to assess the heterogeneity of the microstructure of cement hydration products. Generally, the most important phase is C–S–H, which supplies the highest strength gain to the material, whose structure usually has an amorphous morphology. Besides, portlandite is the other phase present in the cement paste, and its shape is a hexagonal prism. The use of different binders, sample drying preparation, curing temperature, etc. influence the morphology of these phases (Zhang et al. 2018). In this study, the significant formation of the C–S–H phase was observed, as shown in Fig. 19, and Ca(OH)_2_ dispersed throughout the structure of cement paste. A well-developed bond structure was presented in the morphological surface of the designed mortar samples which is compatible with the mechanical properties of the samples.

Observing Fig. 19, the mortar sample having only PC has a relatively dense microstructure and platy Ca(OH)_2_. The replacement of 10% MWTS with PC increased the compactness of the matrix. It can be due to the pozzolanic activity and filling effect of MWTS due to having much smaller particle size (see Fig. 5). Furthermore, it can be emphasized that a large amount of MWTS reacted and converted to C–S–H. However, as the substitution of MWTS was increased, the microstructure worsened. Especially, the increase in the waste product content caused less hydration products and the formation of pores resulting in a relatively loose microstructure which could be due to having lower cement content. Namely, the use of 30% MWTS with FA or BFS as a ternary blend caused the structure to become a porous morphology with a less developed bond phase. Further evidence of pozzolanic reactivity at this curing age was supported by the XRD patterns in Fig. 18, which indicated a higher Ca(OH)_2_ amount in the ternary system. This behavior is in agreement with the mechanical test results. However, some particles of waste products were observed to remain unreacted, suggesting that these particles can act as partially inert material to improve particle packaging (Berry et al. 1994).

## Economic and environmental aspects

As indicated in the previous researches, WTS is a potential pollutant to the environment, so recycling of WTS in cement-based materials provides a new way to upcycle solid waste. Utilization of modified WTS as a binary blend in mortar and the usage of MWTS with FA or BFS as ternary blended binders were investigated in terms of rheological, mechanical, durability, and microstructural aspects. It is worth to say that the reutilization of these waste products into cement-based materials provides disposal of waste without harming the environment and also minimizes the cement content which promotes the environmental sustainability of mortars. However, environmental evaluation is also an important aspect for the development of cement-based materials blended with various waste products. Within this scope, the environmental impacts were analyzed in terms of CO_2_ footprint and embodied energy consumption, while the economical impacts were analyzed in terms of cost for each designed mortar having waste products as binder as shown in Table 6. The CO_2_ emission of water is very low so it is not taken into consideration (Maddalena et al. 2018). In the literature, the emission associated with PC is 0.8–1.0 CO_2_-eq/kg (Scrivener et al. 2018; Flower and Sanjayan 2017). In the current study, it was used as 0.83 based on the study by Ruviaro et al. (2021). Some researchers in earlier studies (Panesar et al. 2019; O’Brien et al. 2009) noted that the CO_2_ emission of FA is negligible because it is a waste by-product of coal-burning power plants, but in the current study, the CO_2_ emission of FA was used as per the study by Yu et al. (2021). Similarly, raw WTS contributes zero CO_2_ emission, as it is also a waste product in WWTPs (Müller et al. 2014; Maddalena et al. 2018). However, in the current study, due to the calcination (0.088 CO_2_-eq/kg) and grinding (0.002 CO_2_-eq/kg) processes, an emission of 0.091 CO_2_-eq/kg was adopted (Ruviaro et al. 2021). The cost and embodied energy used for the production of MWTS were analyzed according to its recycling process, namely, drying, grinding, and calcination. These values were used in the study by Spat Ruviaro et al. (2023). The cost and emission related to the transportation of the raw materials were not considered in the current study. But, it is clear that if an economically and environmentally advantageous cement-based composite is to be produced, these raw materials must be procured from the nearby regions. The impacts of each raw material are summarized in Table 6.

**Table 6:** Unit cost, CO_2_ footprint, and embodied energy of raw materials

	Cost ($/kg)	CO_2_ emission (kg/m^3^)	Embodied energy (MJ/kg)	Ref
PC	0.11	0,83	4.73	(Du et al. 2022; Ruviaro et al. 2021)
FA	0.02	0.008	0.1	(Yu et al. 2021)
BFS	0.1	0.02	1.59	(Du et al. 2022)
MWTS	0.02	0.091	0.97	(Spat Ruviaro et al. 2023; Ruviaro et al. 2021)
Sand	0.02	0.01	0.11	(Du et al. 2022)
Water	0	0	0.01	(Du et al. 2022)
SP	3.6	0.72	18.3	(Du et al. 2022)

### Economical evaluation

**Fig. 20:**
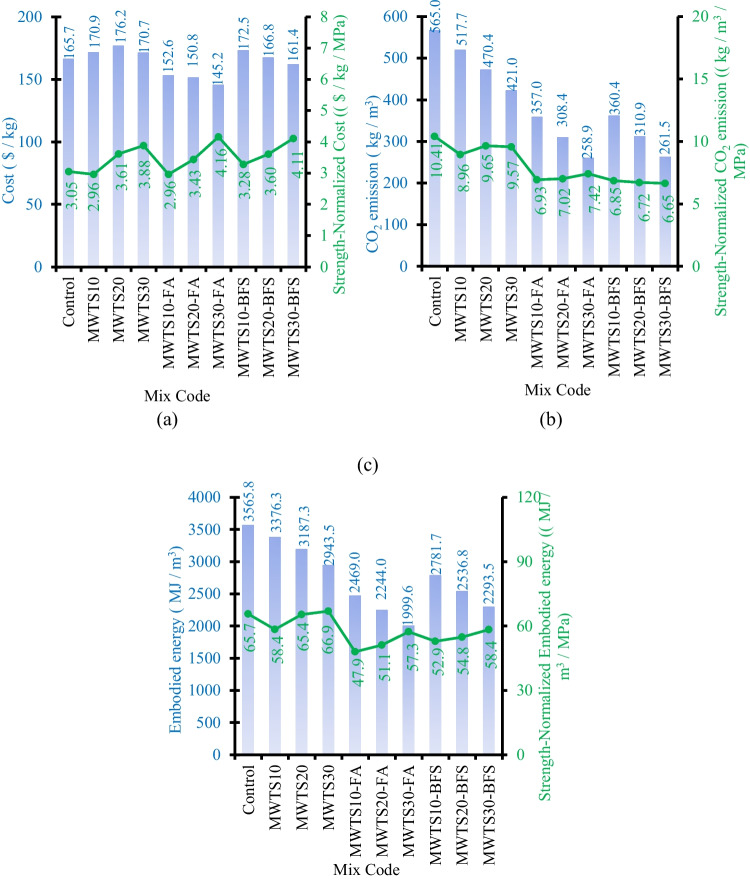
The unit (**a**) cost, **b** CO_2_ footprint, and **c** energy consumption of the designed mortars

The unit cost ($/kg) of each designed mortar with various waste products was calculated by using Eq. 4.

4 $${\text{COST}}= \sum_{i=1}^{n}{c}_{i}{r}_{i}$$

where $${c}_{i}$$ is the unit cost ($/kg) and $${r}_{i}$$ is the mass (kg/m^3^) of the $$i$$-th raw material of the mixture. Figure 20a shows the unit cost of each designed mortar and the 90-day compressive strength-normalized cost values. The inclusion of 10% MWTS, 20% MWTS, and 30% MWTS into control mortar increased the unit cost from $165.7 to $170.9, $176.2, and $170.7, respectively, which is due to the costly price of SP. On the other hand, the addition of 10% MWTS as a binary blend reduced the strength-normalized cost from 3.05 to 2.96 $/m^3^/MPa, while 20% and 30% incorporation of MWTS caused an increase. It indicated that the usage of 10% MWTS as a replacement of cement increased the 90-day compressive strength by a slight increase in cost. In the ternary binder system, the higher price of BFS resulted in higher unit cost values of the designed mortars and thus higher strength-normalized cost values. On the other hand, the incorporation of MWTS with FA decreased the unit cost of the mortars, and, especially, MWTS30-FA achieved the lowest unit cost. However, the strength-normalized cost value of MWTS30-FA was the highest with 4.16 $/m^3^/MPa which indicates its lower 90-day compressive strength relative to other mortars. In addition to this, for MWTS10-FA, the unit cost decreased from $165.7 to $152.6, while the strength-normalized cost also decreased from 3.05 to 2.96 $/m^3^/MPa. This result showed the convenience and advantage of the utilization of 10% MWTS with FA as a ternary binder into the mortar in terms of unit cost and strength-normalized cost.

### CO_2_ footprint

The unit CO_2_ footprint (kg/m^3^) of each designed mortar with various waste products was calculated by using Eq. 5.

5 $${{\text{CO}}}_{2}= \sum_{i=1}^{n}{c}_{i}{r}_{i}$$

where ${c}_{i}$ is the unit CO_2_ emission (kg/m^3^) and ${r}_{i}$ is the mass (kg/m^3^) of the $i$-th raw material of the mixture. The unit CO_2_ emission of each designed mortar and the 90-day compressive strength-normalized CO_2_ emission values are shown in Fig. 20b. As waste product content increased in the binary and ternary blended mortars, the unit CO_2_ emission was reduced sharply due to the reduced cement dosage. As known, the production process of PC releases more than 2 Gt CO_2_ annually. In this sense, the reduction in CO_2_ emission for the cement-based materials having waste products as a binder gave a promising result in terms of environmental aspects. Especially, for the mortars having a high volume of waste product (MWTS30-FA and MWTS30-BFS), the reduction in the unit CO_2_ footprint was approximately 54% relative to the control sample. However, the strength-normalized CO_2_ emission value was reduced from 10.41 to 7.42 kg/m^3^/MPa and 6.65 kg/m^3^/MPa which indicated the lower 90-day compressive strength of the designed mortars. In the binary blended system, 10% MWTS inclusion into the mortar as binder enhanced the 90-day compressive strength and reduced the unit CO_2_ footprint compared to the control sample. However, the inclusion of 20% MWTS into the mortar may be more advisable from an environmental perspective, as it reduces the unit CO_2_ footprint much more (16.74%), although it causes a 10.2% decrease in compressive strength compared to the control sample. On the other hand, in the ternary blended system, the incorporation of 10% MWTS with FA or BFS caused approximately 37% lower unit CO_2_ footprint and 3.13% and 5.16% lower 90-day compressive strength relative to the control sample, respectively. Therefore, it was obvious that the utilization of 10% MWTS with FA/BFS as ternary blended binders was the optimal mixture to reduce CO_2_ footprint by achieving comparable strength.

**Fig. 21:**
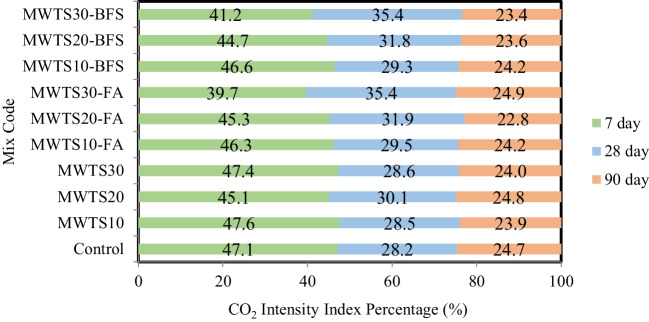
CI percentage for designed samples

The CO_2_ intensity index (CI) was also evaluated, representing the ratio between the CO_2_ emission of material and its compressive strength at a given age (Damineli et al. 2010). It is an important parameter to compare different compositions, cementitious matrices, and binder types. Observing Table 7, CI results for the curing ages of 7, 28, and 90 were calculated, and also, CI percentages with the evolution of ages are shown in Fig. 20. CI is directly related to the resistance gain of material over time. At 7 days, all samples showed lower resistance as seen in Table 7 and Fig. 21. However, as the curing age increased, the compressive strength values increased as a result of the pozzolanic reactions, and thus, CI values were reduced. From Table 7, it is worth to say that all the mortars having various waste products presented lower CI values at all ages due to the ecological efficiency of these mixtures. Besides, the incorporation of FA and BFS with MWTS as a ternary blend reduced the CI values more pronounced and provided the formation of a more eco-efficient composition.

**Table 7:** CO_2_ intensity index (CI) for samples at 7, 28, and 90 curing days

	7 days	28 days	90 days
Control	19.89	11.89	10.41
MWTS10	17.82	10.67	8.96
MWTS20	17.51	11.68	9.65
MWTS30	18.88	11.41	9.57
MWTS10-FA	13.25	8.46	6.93
MWTS20-FA	13.98	9.82	7.02
MWTS30-FA	11.84	10.55	7.42
MWTS10-BFS	13.20	8.30	6.85
MWTS20-BFS	12.74	9.06	6.72
MWTS30-BFS	11.73	10.06	6.65

### Embodied energy

The unit embodied energy (MJ/m^3^) of each designed mortar with various waste products was calculated by using Eq. 6.

6 $$E= \sum_{i=1}^{n}{e}_{i}{r}_{i}$$

where ${e}_{i}$ is the embodied energy (MJ/m^3^) and ${r}_{i}$ is the mass (kg/m^3^) of the $i$-th raw material of the mixture. The unit embodied energy of each designed mortar and the 90-day compressive strength-normalized embodied energy values are shown in Fig. 20c. As expected, a sharp reduction in embodied energy was so prominent for the mortars having waste products as a partial replacement of cement. The production of PC is one of the most energy-intensive processes, causing huge energy consumption so less dosage of cement means lower embodied energy. Regarding binary blended mortars, MWTS10 and MWTS20 caused 5.31% and 10.61% lower unit embodied energy, while the strength-normalized embodied energy of MWTS10 was much more lower than the control sample. For the ternary blended system, due to having higher embodied energy of BFS, the mortars blended with both MWTS and BFS had higher unit embodied energy values relative to the ones with MWTS and FA. Besides, the ternary blend of MWTS and BFS caused higher strength-normalized embodied energy values. Hence, it was found that the combination of MWTS and FA as ternary blend binder reduced unit embodied energy more effectively compared to the other designed mortars. In terms of both embodied energy and strength-normalized embodied energy, the utilization of 10% MWTS with FA as a ternary binder can be advised.

### Comparison of cost and environmental evaluation

**Fig. 22:**
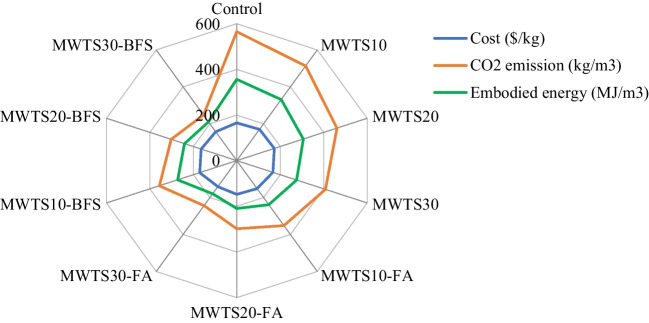
Comparison results of economical and environmental impact of designed samples

The cost and ecological evaluation of 1 m^3^ mortar samples having various waste products are shown in Fig. 22. The aim of this study was to develop a sustainable cement-based material by reducing the cost, CO_2_ footprint, and energy consumption without mitigating the rheological, mechanical, and durability properties. Generally, the enhanced mechanical and durability properties are achieved by higher material cost, energy consumption, and CO_2_ footprint. However, as a novelty, by the recycling of WTS and its usage with other waste products as a ternary binder, the adverse environmental impacts and cost were reduced as given in Fig. 22.

As known, cement has a determinant effect on the cost and environmental impacts of cement-based materials. It was clear that the environmental impact was significantly reduced by recycling WTS and its incorporation with other waste products (FA/BFS). This reason can be attributed to the fact that the utilization of these waste products as a binder lowered the cement dosage resulting in reducing the CO_2_ footprint and energy consumption. Among all designed mortars, it was observed that the utilization of MWTS with FA as a ternary binder into cement-based materials decreased the cost and environmental impacts more specifically. Namely, FA content reduced the CO_2_ footprint by 36.81%, 45.42%, and 54.18% when ternary blended with 10%, 20%, and 30% MWTS, respectively, relative to the control sample. Besides, these values were 30.76%, 37.07%, and 43.92%, for the embodied energy, respectively. This is attributed to the low CO_2_ emission and embodied energy of FA relative to other ingredients, reflecting a lower impact on the total CO_2_ emission and embodied energy of the designed mixtures. Moreover, the cost was reduced by the incorporation of 10%, 20%, and 30% MWTS with FA by 7.93%, 9.01%, and 12.38%, respectively. In addition to these, it should be noted that the cost, CO_2_ emission, and embodied energy of SP were significantly higher than those of the other ingredients of the mixes. Especially, for binary blended mortars with MWTS, the addition of a higher amount of SP to satisfy the desired fresh properties caused an increase in total cost, CO_2_ emission, and embodied energy of the designed mixtures. Due to this reason, minimizing the SP content to achieve an eco-friendly and cost-effective mix was a key factor. As one solution, the utilization of MWTS with BFS as a ternary blend resulted in lower CO_2_ emissions and energy consumption. However, as another solution, a ternary blended system with MWTS and FA reduced the environmental impacts (CO_2_ footprint and energy consumption) and caused a lower cost of cement-based mortar for 1 m^3^. On the other hand, it should be noted that, in the design of eco-friendly, cost-effective, and sustainable cement-based materials, the use of an appropriate amount of MWTS with FA/BFS as ternary binder would become more suitable by achieving comparable rheological, mechanical, and durability properties. From this study, the optimal replacement percentage of MWTS with cement was found as 10% for the ternary systems. It is also worth to say that the supply of waste products should be limited to local. Otherwise, the high cost, CO_2_ emission, and energy consumption caused by the transportation of the wastes would reduce their environmental and economical benefits.

## Conclusion

This study investigated the effect of binary blends of MWTS and ternary blends of MWTS and FA or BFS as a partial substitution of cement by weight on the rheological, mechanical, durability, and microstructural properties of mortars. Based on the experimental study, the following conclusions were obtained:

- The yield stress and viscosity of mortar mixtures increased with the increasing amount of MWTS in the binary blend relative to the control mixture, while the incorporation of 10% and 20% MWTS caused similar rheological properties. On the other hand, the ternary blend of 10% and 20% MWTS with FA or BFS improved the rheological parameters compared to the control mixture having only cement. Namely, the lowest viscosity and yield stress values were obtained from MWTS10-FA with a reduction of 12.92% and 31.63%, respectively.
- The use of 10% MWTS as a binary blend caused 6.4% and 5.4% enhancement for the compressive and flexural strengths at 90 days, respectively, while the increase in MWTS content reduced the strength values at all ages. On the other hand, all ternary blended mortars exhibited lower strengths relative to the control sample for all ages.
- Regarding ternary blended mortars, the incorporation of MWTS with BFS showed better-hardened performance for all ages. Their slightly lower cement index values and lower rate of decrease in strength relative to the control sample indicated that the use of 10% MWTS with BFS as a ternary blend was possible to produce sustainable cement-based materials due to having low cement dosage and the usage of high amount of waste products.
- Based on the MIP test results, the incorporation of 10% MWTS with cement resulted in reduced pore diameter and the lowest porosity among all designed mortars. Besides, the addition of FA or BFS into binary blended MWTS samples increased the volume of large capillary pores resulting in higher porosity. Especially, the mortars with 30% MWTS in ternary systems caused a lower fraction of gel pores which reveals the formation of less hydration products and the presence of a large number of unreacted particles.
- The utilization of MWTS as a binary blend and MWTS with FA/BFS as a ternary blend into mortar reduced the Ca(OH)_2_ peak of the mortar samples. Their pozzolanic activity makes these waste products’ usage as binary and ternary into cement-based materials possible. However, due to having lower intensity of alite, belite, and Ca(OH)_2_, it can be emphasized that the use of MWTS with BFS as a ternary blend became more effective in the process of hydration reactions of cementitious materials.
- The use of 10% MWTS as a binary blend improved the compactness of the microstructure by reducing the porosity but increasing the dosage of MWTS worsened the microstructure due to the less developed bond phase. Even, the ternary use of 30% MWTS with FA or BFS caused the structure to become porous morphology.
- Considering the economical and environmental impacts of mortars with various waste products, the ternary use of MWTS and FA demonstrated more significant benefits from the perspective of developing eco-friendly, cost-effective, and sustainable cement-based materials.

Consequently, this research indicated that the use of 10% MWTS as a binary blend into mortar was beneficial to improve the mechanical, durability, and microstructural properties. However, as a key finding, the incorporation of MWTS with BFS or FA suppressed the disadvantage of the use of recycled WTS as a binary binder in terms of rheological parameters and environmental and economical aspects with comparable strength values. Therefore, it should be noted that, in the design of eco-friendly, cost-effective, and sustainable cement-based materials, the use of the appropriate amount of MWTS with FA/BFS as a ternary binder would become more suitable by achieving comparable rheological, mechanical, and durability properties. Especially, ternary blended binder system with 10% MWTS and 30% FA reduced the environmental impacts (CO_2_ footprint and energy consumption) and caused a lower cost of cement-based mortar for 1 m^3^ more effectively, with comparable mechanical and durability properties. From this study, the optimal replacement percentage of MWTS with cement was found as 10% for the ternary systems after testing the mortars binary/ternary blended with 10%, 20%, and 30% MWTS. The use of these waste products as a binder in a ternary system as a replacement for cement conserves the environment by saving large amounts of waste products and helps reduce a carbon footprint. Nevertheless, as a future recommendation, the effects of ternary usage of MWTS with FA/BFS should also be investigated by adding different amounts of FA/BFS and also with different MWTS dosages. More comprehensive experimental work can be planned by the incorporation of different percentages of MWTS and FA/BFS using the findings of the current study and, thus, different optimum values can be established. Moreover, the long-term performance of WTS blended mortars under a range of environmental stresses such as sulfate attack, chloride ingress, freeze-thaw deterioration, and carbonation should be assessed.

## Data Availability

Not applicable.
